# Novel vanadium-dependent haloperoxidases from macroalgae and their expression in response to biotic and abiotic stressors in *Saccharina latissima*

**DOI:** 10.1007/s42995-026-00367-4

**Published:** 2026-03-30

**Authors:** Timo Jensen, Mira Wilkens, Holger Rehmann, Maria Mucke, Dominik Bents, Petra Janning, Andreas Brockmeyer, Stefan Veltel, Matthias Peipp, Antje Labes, Wolfgang Bilger, Levent Piker

**Affiliations:** 1 Coastal Research and Management GbR, 24159 Kiel, Germany; 2 Plant Ecophysiology, Christian-Albrechts-University Kiel, 24118 Kiel, Germany; 3 University of Applied Sciences Flensburg, 24943 Flensburg, Germany; 4 Faculty of Nature and Engineering, City University of Applied Sciences Bremen, 28199 Bremen, Germany; 5 Max Planck Institute of Molecular Physiology, 44227 Dortmund, Germany; 6 Division of Antibody-Based Immunotherapy, Department of Internal Medicine II, University Medical Center Schleswig-Holstein and Christian-Albrechts-University Kiel, 24105 Kiel, Germany

**Keywords:** Vanadium-dependent haloperoxidases, Algae, Stress, *Saccharina latissima*, Transcriptomics, Gene expression

## Abstract

Vanadium-dependent haloperoxidases (vHPOs) are essential enzymes in macroalgae, known for their ability to catalyze halogenation reactions using halides and hydrogen peroxide. These enzymes facilitate the biosynthesis of halogenated compounds that contribute to the defense mechanisms of algae and are considered to play a crucial role in the oxidative stress response. Using a transcriptomic approach, we discovered and analyzed about 70 novel vHPO sequences from nine different macroalgae species collected in the Baltic Sea. All sequences were carefully selected for their universal catalytic center characterized by structurally conserved residues essential for catalysis and vanadate-binding. Mass spectrometry provided evidence for eight of those proteins in extract fractions of the brown alga *Saccharina latissima*. Using RT-qPCR, we also investigated the role of vHPO gene expression in stress response on a subset of *S. latissima* vHPO transcripts by exposing it to different stressors including copper excess, oxidative stress, and biotic stress mimicked by elicitor treatment using a *S. latissima* tissue homogenate. The optimal quantum yield of photosystem ll served as a physiological plant stress parameter (*F*_V_/*F*_M_). Our data support the hypothesis that these enzymes are involved in mitigating oxidative stress, as suggested by up-regulation in gene expression following H_2_O_2_ and elicitor treatments, and are likely involved in environmental stress response. This work advances our understanding of vHPO function in marine algae and highlights their potential role in responding to environmental stress.

**Supplementary Information:**

The online version contains supplementary material available at 10.1007/s42995-026-00367-4.

## Introduction

Plants and algae are constantly exposed to various environmental stimuli, which can be of biotic and abiotic nature. Some of these stimuli may negatively impact the organism’s growth, development, or productivity and are referred to as stress (Buchanan 2000). These organisms have developed various mechanisms to combat stress by sensing the stressors and mediating an appropriate biochemical and physiological plant response (Verma et al. 2013). The mediative response is a complex signaling network that cumulates in transcriptional changes permitting stress tolerance (Verma et al. 2013). These changes may concern transcription, translation, metabolism, protein activity and, possibly, programmed cell death (Sewelam et al. 2016). Important signal molecules during a stress response are reactive oxygen species (ROS) (Apel and Hirt 2004; Mittler et al. 2004; Kawano 2003; Sewelam et al. 2016), which include singlet oxygen (^1^O_2_), superoxide (O_2_^•−^) and hydrogen peroxide (H_2_O_2_). ROS are continuously produced and degraded during normal aerobic metabolism, but rapidly produced under stress conditions in high concentrations (Apel and Hirt 2004; Miller et al. 2009; Sewelam et al. 2016). This is referred to as oxidative stress (Sies 1991), which is cytotoxic as ROS can damage proteins, DNA, and lipids (Apel and Hirt 2004). To regulate ROS signaling, plants and especially algae have developed enzymatic and non-enzymatic antioxidative systems to detoxify ROS (Apel and Hirt 2004). One of the main enzyme classes with antioxidant capacity is peroxidase (E.C. 1.11.1.x). It is found in all kingdoms (Passardi et al. 2007) and exhibits a large variety of functions in the plant life cycle (Pandey et al. 2017), with one of them being the reduction of H_2_O_2_ and other peroxides (Bindoli and Rigobello 2013).

This class of enzymes includes various subclasses with unique functions and cofactor dependencies (Pandey et al. 2017; Passardi et al. 2007). One particularly intriguing subclass is the haloperoxidases. These enzymes exhibit the remarkable ability to catalyze halogenation reactions, making them subjects of interest for green chemistry and pharmaceutical applications (Renirie et al. 2008; Wever et al. 2018). Vanadium-dependent haloperoxidases (vHPOs) contain vanadate as a prosthetic group (Manley 2002; Wever et al. 2018). Among these, vanadium-dependent chloroperoxidases (vCPOs) are capable of oxidizing chloride, bromide and iodide, while vanadium-dependent bromoperoxidases (vBPOs) can use both bromide and iodide, and vanadium-dependent iodoperoxiases (vIPOs) are specific to iodine (Colin et al. 2003). The latter two, vBPOs and vIPOs, are found primarily in marine algae (Colin et al. 2005; Jordan et al. 1991), where they are assumed to be involved in cell wall assembly, protection against oxidative stress, and chemical defense (Leblanc et al. 2006, 2015). They play a key role in uptake of bromide and iodide from seawater and formation of halogenic compounds, leading to the highest known accumulations of iodine in marine macroalgae, especially in Laminariaceae, compared to any other living species (Küpper et al. 1998; Verhaeghe et al. 2008b; Wever et al. 2018). In terms of environmental stress responses, the formed halogenated compounds are bioactive against herbivore and pathogen attack (Manley 2002; Potin et al. 2002), which extends the functional specialization of vHPOs during pathogenic invasion. Furthermore, it is assumed that during Cu^2+^ stress, released iodide may form complexes with Cu^2+^, leading to a non-bioavailable form of copper (Ritter et al. 2010), which might have a beneficial effect against Cu^2+^ excess. However, the underlying mechanisms of vHPO-mediated stress responses remain underexplored as many vHPO genes in marine algae are still unidentified.

In this work, we used a proteo-transcriptomic approach for the discovery of novel vHPOs from a variety of macroalgae of the Baltic Sea, harnessing bioinformatic tools to predict their features and characteristics. In order to de-monstrate the involvement of vHPO in stress mitigation, we applied biotic and abiotic stressors to the sublittoral, perennial brown alga *Saccharina latissima.* RT-qPCR experiments based on the obtained sequences were used to explore the vHPO stress response. Three stressors, which were expected to induce stress responses in *S. latissima*, were chosen: abiotic stress in the form of excess copper (Cu^2+^), biotic stress induced by a *S. latissima* homogenate-based elicitor treatment, and direct application of oxidative stress in the form of H_2_O_2_. To assess the extent of stress effects in the treated algae, the optimal quantum yield *F*_V_/*F*_M_ of the photosystem II (PS II) was recorded over the course of the experiment. Our studies give insights into the biodiscovery of novel proteins from algae, with a particular emphasis on vHPOs, and their putative roles in stress response.

## Materials and methods

### Sampling

For transcriptome assembly, algae samples were harvested by snorkeling in the coastal waters of the Baltic Sea in Kiel, Germany. Harvesting months, location, and environmental conditions (water temperature, pH, salinity, and dissolved oxygen) are provided in Table 1. Each specimen was carefully detached from its substrate to preserve the holdfast, quick-frozen on dry ice, and stored at − 80 °C until use.

**Table 1:** Overview of algal species sampled for transcriptome analysis, specifying the harvesting month and location as well as the environmental parameters measured during collection

Species	Harvesting month	Harvesting location	Water temperature (°C)	pH	Salinity (‰)	Dissolved oxygen (mg/L)
*Fucus vesiculosus*	Nov 2020	54°22′08.3"N 10°08′49.9"E	10.2	7.91	17.95	8.05
*Saccharina latissima* (Sample 1)	Nov 2020	54°22′08.2"N 10°08′50.1"E	10.2	7.91	17.95	8.05
*Ceramium virgatum*	Dec 2020	54°28′48.7"N 10°03′55.4"E	8.2	8.00	18.78	9.10
*Bryopsis plumosa*	Dec 2020	54°28′48.7"N 10°03′55.4"E	8.2	8.00	18.78	9.10
*Fucus serratus*	Dec 2020	54°28′48.7"N 10°03′55.4"E	8.2	8.00	18.78	9.10
*Phyllophora crispa*	Dec 2020	54°28′48.7"N 10°03′55.4"E	8.2	8.00	18.78	9.10
*Ahnfeltia plicata*	Feb 2021	54°27′21.8"N 10°11′35.9"E	1.6	8.67	15.29	12.87
*Furcellaria lumbricalis*	Mar 2021	54°27′21.8"N 10°11′35.9"E	5.6	9.61	14.92	15.20
*Chorda filum*	Apr 2021	54°22′07.1"N 10°08′02.8"E	7.5	9.07	12.91	11.53
*Saccharina latissima* (Sample 2)	Apr 2021	54°22′09.7"N 10°08′38.7"E	6.9	9.14	12.00	14.90

For stress experiments, *S. latissima* sporophytes of 30 to 100 cm length were collected in April 2022 in the Baltic Sea in Kiel (54°22′09.7"N 10°08′38.7"E), Germany, and transported in seawater. To minimize effects of collection and transport, the thalli were acclimated under culturing conditions for 48 h. The thalli were cultured in a climate cabinet (CLF Plant Climatics, Wertingen, Germany) using 28 cm × 20 cm × 14 cm polypropylene vessels, each containing 3.5 L of seawater filtered through a 10 µm mesh. Controlled conditions with 15 °C, aeration with air (approx. 170 mL/min), 35 µmol photons m^−2^ s^−1^, and a 12:12 h day–night cycle (with two-hour gradual progression) were applied. Light was provided by fluorescent tubes (Super TL-D 18W/840, Phillips, Hamburg, Germany). At the end of the acclimation phase, the algae were randomly distributed to different treatment groups. The thalli were tagged with identification numbers that were attached to the rhizoid using a labeled thread.

### Transcriptome generation and identification of vHPO genes

Transcriptomes were generated from all algae species in Table 1. RNA was extracted from one half of the samples (*Fucus vesiculosus*, *Ceramium virgatum*, *Bryopsis plumosa*, *Fucus serratus*, and *Phyllophora crispa*) by Microsynth AG, Balgach, Switzerland. The other half (*Ahnfeltia plicata*, *Furcellaria lumbricalis*, *Chorda filum*, and *Saccharina latissima* (Sample 2)) was extracted using the modified CTAB method described by Jensen et al. (2023) and sequenced by StarSEQ GmbH, Mainz, Germany. *Saccharina latissima* (Sample 1) was both extracted and sequenced by StarSEQ. Both sequencing service providers used the RNeasy Plant Mini Kit (Qiagen N.V., Venlo, Netherlands) for RNA extraction, followed by poly(A) enrichment, cDNA synthesis, and Illumina library preparation (New England Biolabs, Inc., Ipswich, USA). Sequencing was performed on an Illumina NextSeq 500 platform (Illumina, Inc., San Diego, USA), generating 150 bp paired-end reads.

**Fig. 1:**
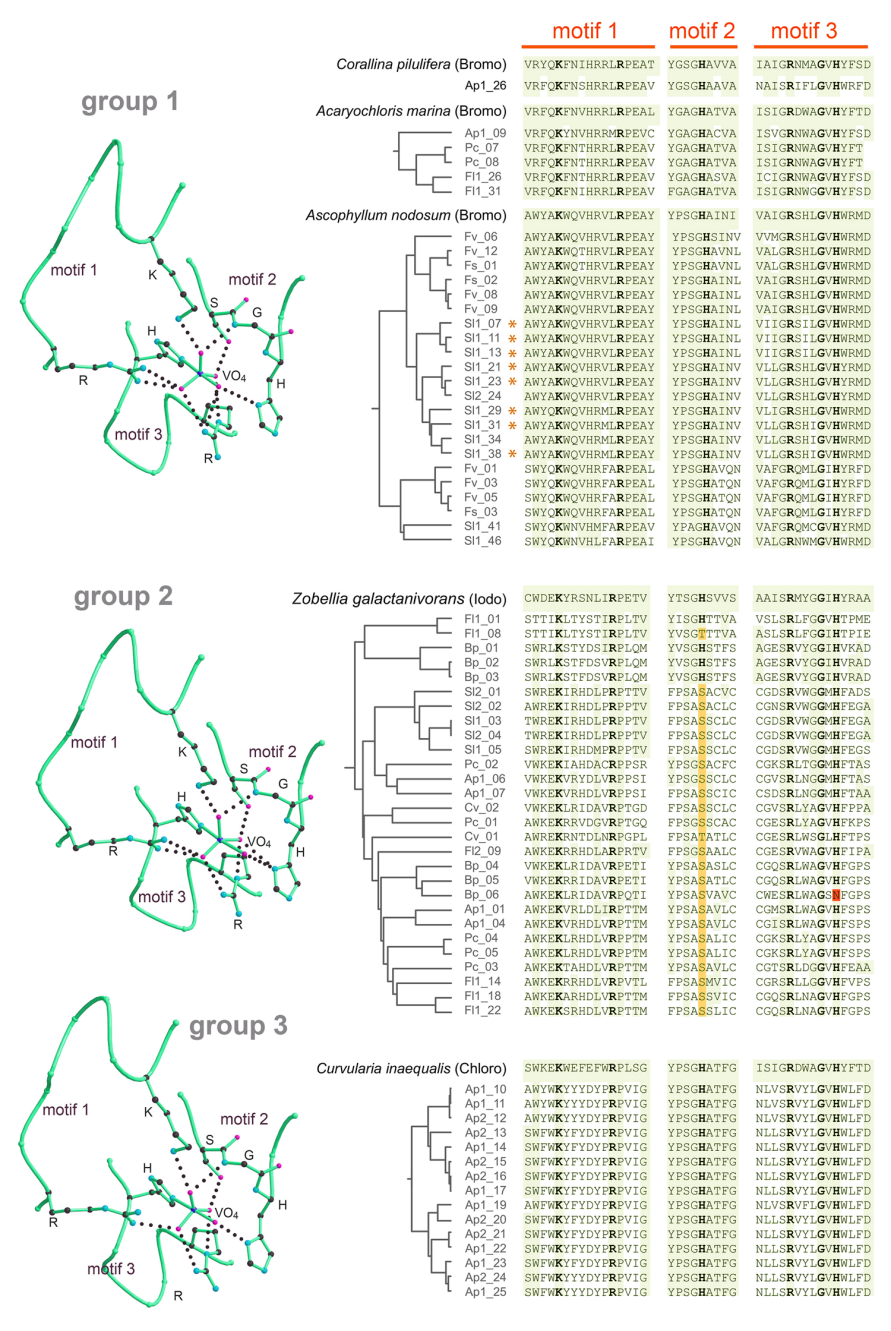
Identification of putative vHPOs. Crystal structures of vanadate bound vHPOs from *A. nodosum* (group 1; pdb: 5AA6), *Z. galactanivorans* (group 2; 4CIT), and *C. inaequalis* (group 3; 1IDQ). Vanadate-binding regions are shown as backbone traces with Cα atoms; key residues are in ball-and-stick representation with hydrogen bonds as dotted lines. Putative vHPOs were assigned to the closest structurally characterized vHPO and aligned with Clustal Omega (Sievers et al. 2011). Residues involved in vanadate-binding are highlighted in bold. Residues identical to the closest known vHPO are shaded; substitutions at vanadate-binding positions are yellow or red. vHPOs from *S. latissima* marked with asterisk (*) were detected by MS/MS in active protein fraction. Ap: *Ahnfeltia plicata*, Bp: *Bryopsis plumosa*, Cv: *Ceramium virgatum*, Fv: *Fucus vesiculosus*, Fs: *Fucus serratus*, Fl: *Furcellaria lumbricalis*, Pc: *Phyllophora crispa*; Sl: *Saccharina latissima*

The obtained sequence data were processed with fastp (Chen et al. 2018), normalized with BBNorm (Bushnell et al. 2017), and assembled with rnaSPAdes (Bushmanova et al. 2019). The obtained sequences were six-frame translated with sixpack (EMBOSS) requesting a minimal read length of 100 amino acids and a methionine as start. Putative vHPOs were identified by searching the obtained sequences with known vHPOs with BLAST 2.12. (Altschul et al. 1990). Sequences of known vHPOs were taken from the Protein Data Bank (pdb; Berman et al. (2000)) and reflect structurally and biochemically well-characterized proteins from *Corallina pilulifera* (pdb entry 1UP8), *Acaryochloris marina* (5LPC), *Ascophyllum nodosum* (5AA6), *Zobellia galactanivorans* (4CIT), and *Curvularia inaequalis* (1IDQ). The crystal structures of these vHPOs were superimposed in Bragi (Schomburg and Reichelt 1988) and structural representations (Fig. 1) were generated with Molscript (Kraulis 1991) and Raster3D (Merritt and Murphy 1994). The resulting hits were manually curated by checking the sequence data for plausibility and removing duplicate sequences from different sequence runs of the same algal species. For analysis purposes, sequence reads were back-mapped to the assemblies with Minimap2 (Li 2018) and inspected with Integrative Genomics Viewer (Robinson et al. 2011).

### Protein extraction and purification

All steps were performed on ice unless otherwise stated. Frozen *S. latissima* tissue was dispersed in ice-cold homogenization buffer (100 mmol/L Tris–HCl, 2 mmol/L CaCl₂, 1 mmol/L dithiothreitol (DTT), 0.01% Triton X-100, 5% glycerol, pH 9) using an UltraTurrax at max. speed (IKA-Werke, Staufen, Germany) for 5 min (1 min intervals with 30 s breaks). After the addition of 2% polyvinyl polypyrrolidone (PVPP) and incubation for 20 min, cell debris and PVPP were removed by centrifugation (9000 *g*, 20 min, 4 °C). The crude extract was further centrifuged (25,000 *g*) and proteins were precipitated with 4 volumes of acetone (− 20 °C) for 12 h. The pellet was suspended in 50 mmol/L Tris–HCl buffer (pH 9) and insoluble material was removed by centrifugation (16,000 *g*). Algal proteins were purified by anion exchange chromatography (AEX) followed by size-exclusion chromatography (SEC) on an FPLC system (NGC, Bio-Rad Laboratories, Hercules, CA, USA) at a flow rate of 1.5 mL/min. Protein solutions were applied to an AEX column (EconoFit UNOsphere Q, Bio-Rad Laboratories, Hercules, CA, USA) equilibrated with 50 mmol/L Tris–HCl (pH 9). After washing with 12 column volumes (CV) of equilibration buffer, proteins were eluted stepwise with 5 CV of NaCl (50 to 1000 mmol/L) in 50 mmol/L Tris–HCl (pH 9). Fractions were assayed for peroxidase activity, pooled, concentrated and separated by SEC (HiLoad® 16/600 Superdex® 200 pg, Cytiva, Marlborough, MA, USA). Active fractions were concentrated on centrifugal filters (Amicon® Ultra, Merck KGaA, Darmstadt, Germany) and analyzed by native polyacrylamide gel electrophoresis (NP).

### In-solution peroxidase activity assays

Peroxidase activity of protein fractions was measured by bromination of thymol blue with bromide, based on a modified method from Verhaeghe et al. (2008a). Peroxidase reactions were performed in 50 mmol/L potassium phosphate buffer (pH 7.5) containing 0.25 mmol/L thymol blue, 20 mmol/L potassium bromide, and the sample of interest. Reactions were initiated with 10 mmol/L H₂O₂, and monitored at 620 nm over a 10-min period at 30 °C in 30 s intervals using a SpectraMax 340 PC (Molecular Devices, San Jose, CA, USA). Enzyme activities were calculated using the Lambert–Beer law (*ε* = 37.2 mM⁻^1^ cm⁻^1^). One unit (U) was defined as the amount of enzyme required to catalyze the formation of 1 nmol of TBBr_2_ per min under defined assay conditions.

### Native PAGE and in-gel activity assays

NP was performed on Tris–glycine gels following Green (2012, p. 1602), without sodium dodecyl sulfate (SDS). Electrophoresis was conducted in 1 × NP running buffer (25 mmol/L Tris–HCl, 192 mmol/L glycine) at 120 V for 60 min at 4 °C using a Mini-PROTEAN® 3 Cell (Bio-Rad Laboratories, Hercules, CA, USA). To determine in-gel peroxidase activity, the NP gels equilibrated in 100 mmol/L potassium phosphate buffer (pH 7.5) for 15 min and stained in the same buffer with 20 mmol/L potassium bromide, 0.25 mmol/L thymol blue, and 10 mmol/L H₂O₂. Active bands appeared after 5–10 min and were excised for mass spectrometric (MS) analysis by in-gel trypsin digestion.

### MS sample preparation

In-gel digestion for MS analysis was performed according to Shevchenko et al. (2006) with modifications. All mixing and incubation steps were performed on a Thermomixer comfort (Eppendorf SE, Hamburg Germany). Briefly, gel pieces were stepwise treated with 200 µL of fixation solution (10% (v/v) acetic acid in 40% (v/v) ethanol, 16 h, RT), 200 µL of washing solution 1 (25 mmol/L aqueous ammonium bicarbonate solution (AmBic) in acetonitrile (ACN; 3:1, v/v), 30 min, 600 rpm, 37 °C), and 200 µL of washing solution 2 (25 mmol/L AmBic in ACN (1:1, v/v), 15 min, 600 rpm, 37 °C). Following reduction (200 µL of 50 mmol/L DTT in 25 mmol/L AmBic, 45 min, 37 °C) and alkylation (200 µL of 55 mmol/L iodoacetamide in 25 mmol/L AmBic, 1 h, in the dark), gel pieces were again washed with washing solution 2 (200 µL, 15 min, 25 °C). After removal of washing solution 2, 100 µL of ACN (100%) was added to dehydrate gel pieces. Afterward, ACN was replaced by 75 µL digestion solution (13 ng/µL trypsin in 25 mmol/L AmBic), which was allowed to be absorbed by the gel pieces (15 min, 600 rpm, RT). Then, 75 µL of 25 mmol/L AmBic was added and tryptic digest was carried out overnight (350 rpm, 37 °C). Digestion was stopped with 10 µL of 10% (v/v) trifluoroacetic acid (TFA) followed by incubation on ice for 30 min in a sonication bath (Ultrasonic Cleaner, VWR International, Radnor, PA, USA). Peptides were extracted twice with 75 µL ACN, (15 min each, 350 rpm, 25 °C) pooled and dried in a vacuum concentrator (RVC 2–18, Martin Christ Gefriertrocknungsanlagen GmbH, Osterode am Harz, Germany) at RT. Samples were reconstituted in 20 µL of equilibration buffer (0.5% TFA in 5% ACN) and peptides were concentrated using C18 spin columns (Pierce™ C18 Spin Columns, Thermo Fisher Scientific Inc., Waltham, MA, USA).

### Mass spectrometry analysis by nano-HPLC/MS/MS

Tryptic peptides were analyzed by nano-HPLC–MS/MS using an Ultimate™ 3000 RSLC nano-HPLC system and a Q Exactive™ HF Hybrid Quadrupole-Orbitrap mass spectrometer equipped with a nano-spray source (all ThermoFisher Scientific). Briefly, the lyophilized tryptic peptides were dissolved in 10 µL 0.1% TFA and 9 µL of these samples was loaded onto a C18 PepMap 100 column (5 µm, 100 Å, 300 µm ID * 5 mm, Dionex, Germany) and enriched using 0.1% TFA and a flow rate of 30 µL/min for 5 min. Subsequently, the peptides were separated on a C18 PepMap 100 nano-column (3 µm, 100 Å, 75 µm ID * 50 cm) using a linear gradient from 5.0% to 30.0% solvent B in 30 min with a flow rate of 300 nL/min (solvent A:water with 0.1% formic acid, solvent B:acetonitrile with 0.1% formic acid). The nano-HPLC was online coupled to the mass spectrometer using a standard Tip emitter (Tip-ID 10 μm). A mass range of m/z 300 to 1650 was acquired with a resolution of 120,000 for full scan, followed by up to ten high-energy collision dissociation (HCD) MS/MS scans of the most intense at least doubly charged ions with a resolution of 30,000.

Protein identification and iBAQ calculation were performed using MaxQuant v.2.4.1.4 (Cox and Mann 2008), including the Andromeda search algorithm. Mass spectrometry data were analyzed using six-frame ORF databases generated from each of the two *S. latissima* transcriptomes as reference. Searches allowed full tryptic cleavage with up to two miscleavages. Carbamidomethylation (fixed) and oxidation of methionine and N-terminal acetylation (variable) were included. The mass accuracy was set to 20 ppm for the first and 7 ppm for the second search. The false discovery rates for peptide and protein identification were set to 0.01. Only proteins for which at least two peptides were quantified were considered for further analysis.

### Bioinformatic characterization of selected vHPO-encoding contigs

Obtained putative vHPO sequences were subjected to a pipeline of different analysis tools. Molecular mass and isoelectric point were calculated with ‘ipc’ (v1.0, Kozlowski (2016)) using the Bjellqvist method (Bjellqvist et al. 1993). Protein function was predicted with ‘InterProScan’ (v5.52–86.0, Jones et al. (2014)). For signal peptide prediction and potential subcellular localization, ‘HECTAR’ (v1.3, Gschloessl et al. (2008)) was used for brown algae, whereas ‘signalP’ (v5.0b, Almagro Armenteros et al. (2019a)), ‘targetP’ for plants (v2.0, Almagro Armenteros et al. (2019b)), and ‘deeploc’ (v1.0, Almagro Armenteros et al. (2017)) with BLOSUM62 protein encoding were used for red and green algae. All sequences were checked for the presence of a Kozac consensus sequence at the start of the ORF (RxxATGG). Amino acid sequences were blasted against NCBI Genbank using blastp (Altschul et al. 1990).

### Stress experiments

Stress experiments consisted of a treatment and a recovery phase. The time points were defined as follows: *t*_0_ (before treatment), *t*_1_ (immediately after treatment), *t*_2_ and *t*_3_ (after 4 h and 24 h recovery, respectively). Due to the different treatment durations (reported below), the treatments were staggered to be terminated simultaneously at *t*_1_. To initiate recovery, the thalli were rinsed and placed in fresh and filtered seawater.

To assess the sub-lethal concentrations of Cu^2+^ and H_2_O_2_, preliminary experiments were performed by applying serial dilutions of the stressors. Three acclimatized thalli of *S. latissima* were cut in 2 cm × 2 cm pieces, acclimated for 2 h and distributed into Petri dishes containing 50 mL of filtered seawater (5 pieces/dish). Cu^2+^ or H_2_O_2_ was applied at concentrations ranging from 0 to 20,000 µg/L and 0 to 7 mmol/L H_2_O_2_, respectively. After 6 h for Cu^2+^ and 0.5 h for H_2_O_2_, the thalli were transferred to fresh seawater for recovery. *F*_V_/*F*_M_ was measured for each thallus piece at *t*_0_–*t*_3_ and additionally after 13 h of recovery.

For the main stress experiment, Cu^2+^ stress was applied using a final concentration of 300 µg/L Cu^2+^ as copper sulfate for a period of 6 h. Direct oxidative stress was induced by a final concentration of 3 mmol/L H_2_O_2_ for 30 min, while biotic stress was simulated using 230 mL of a *S. latissima* homogenate-based elicitor solution for 6 h. This solution was produced by homogenizing 43 g of fresh tissue of *S. latissima* in 200 mL filtered seawater using an Ultra Turrax (TP 20,000 min^−1^, IKA-Werke GmbH, Staufen, Germany) for five minutes until tissue pieces with a maximum size of approximately 1 mm were achieved and the solution took on a brownish color. Each treatment was applied in 3.5 L of filtered seawater; untreated seawater served as control.

The experiment was performed in biological triplicates, which were kept in separate vessels and consisted of two thalli each. At each time point, a half thallus from each replicate was harvested for transcriptomic analysis by cutting the thalli longitudinally at *t*_0_ and *t*_2_, with the second half collected at *t*_1_ and *t*_3_. The samples were rinsed in distilled water, frozen in liquid nitrogen, crushed for homogenization, and stored at − 80 °C until further analysis.

### Measurement of *F*_V_/*F*_M_

The plant stress parameter *F*_V_/*F*_M_ was measured at each time point using an imaging pulse-amplitude-modulation (PAM) chlorophyll fluorometer (Walz GmbH, Effeltrich, Germany) (Schreiber 1983). Before each measurement, the thalli were kept in darkness for 10 min. *F*_0_ and *F*_V_/*F*_M_ were averaged over a rectangular area (approx. 15 cm × 13 cm or less if the thallus was smaller) of the thallus base. Stress-induced PSII damage was calculated as a percentual reduction of *F*_V_/*F*_M_ from *t*_0_ to the following time points, respectively, corrected by the respective control. Test measurements were performed on the elicitor solution as well as on a freshly immersed thallus pieces to ensure that measured stress effects were not caused by potential residues of the elicitor solution.

### Real-time quantitative polymerase chain reaction (RT-qPCR)

***RNA extraction*** RNA was extracted from *S. latissima* samples using a modified procedure of the CTAB method as described by Jensen et al. (2023). Polyphenolic separation steps were performed by centrifuging at 12,000 *g* and the second separation step was performed with chloroform/isoamyl alcohol (C/I) [24:1] (ROTI®-C/I, Carl Roth GmbH, Karlsruhe, Germany) instead of phenol/chloroform/isoamyl alcohol [25:24:1] (ROTI®-Aqua-P/C/I) for a better removal of phenolic residues as suggested by the authors. RNA quality was examined using a NanoDrop spectrophotometer (Thermo Fisher Scientific GmbH, Waltham, Massachusetts, USA). RNA extracts that revealed *A*_260nm_/*A*_230nm_ ratios below 1.9 were treated with an RNA clean-up kit (RNA Clean and Concentrator-100, Zymo Research Europe GmbH, Germany) or re-extracted until a good RNA quality was achieved. RNA integrity was tested for a random subset of 12 samples using the Agilent 2100 Bioanalyzer System (Plant RNA Nano assay, Agilent Technologies, Waldbronn, Germany) according to the manufacturer’s instructions.

***Gene expression analysis*** 500 ng RNA per sample was reverse-transcribed into cDNA using the QuantiTect® Reverse Transcription Kit (QIAGEN GmbH, Hilden, Germany) with half the volumes as suggested by the manufacturer’s instructions (QIAGEN GmbH, 2009). Primer sets for four vHPO genes from *S. latissima* were designed using Primer-BLAST (Ye et al. 2012), targeting high-confidence, less conserved sequence regions from the assembled contigs (Supplementary Table S1). As reference genes, *eukaryotic translation initiation factor 5B (EIF5B*) and *NADH dehydrogenase (ubiquinone) subunit 11* (*NDH*) were considered, and their qPCR primer sequences were used unmodified from Xing et al. (2021). All primer pairs were evaluated for target specificity by performing Primer-BLAST searches against both *S. latissima* transcriptomes to minimize the risk of nonspecific binding. Additionally, target specificity was confirmed through melting curve analysis (∆1 °C/5 min) at the end of the qPCR cycle and by examining the qPCR products via gel electrophoresis on a 2% agarose gel to verify the expected product sizes.

Both reference genes were analyzed across all samples of the stress experiment and their Cq values were checked for correlation (Pearson's correlation coefficient = 0.84, *P* < 0.001). Due to lower Cq variability, *EIF5B* (range: 19.4–24.1; Δ = 4.7) was selected over *NDH* (range: 18.2–25.9; Δ = 7.7) as reference gene.

Reverse transcriptase quantitative polymerase chain reaction (RT-qPCR) was conducted using the Rotor-Gene SYBR Green PCR Kit (QIAGEN GmbH, Redwood City, California, USA) with the Rotor-Gene Q PCR Cycler (QIAGEN GmbH) according to the manufacturer’s instructions. Amplification and melting curves were generated using Rotor-Gene Q (version 2.3.4 Build 3, QIAGEN GmbH). The threshold was manually set to 0.038, where the fluorescence increased exponentially and exceeded the background signal. Run-to-run variability was controlled by technical duplication of selected samples. Relative changes in gene expression were calculated according to the 2^−ΔΔCq^ method (Livak and Schmittgen 2001) as *x*-fold changes to the appropriate control treatment samples and to the *t*_0_-sample from the same treatment group, respectively.

### Data analysis and statistics

Statistics was performed using R (version 4.2.1), implemented in RStudio (build 2022.07.2, Posit PBC, Boston, USA). Normality of data and normality of residues were tested by Shapiro–Wilk test and homogeneity of variances was tested by *F*-test. To test for significant differences in the stress experiment, one-way ANOVAs with subsequent Dunnett´s test [R package DescTools, version 0.99.48, Signorell (2014)] were conducted for each treatment group individually. For fold change (to control) and optimal quantum yield, *t*_0_ of the corresponding treatment was used as a reference in Dunnett´s test, whereas for fold changes (to *t*_0_), the treatments were compared to the control of the respective time point. Due to the exponential nature of the Cq data, only values transformed to 2^−Cq^, 2^−ΔCq^ and 2^−ΔΔCq^ (= fold change) were statistically evaluated. Sources of variability were analyzed within ΔCq values by Mann–Whitney U test and within the specific activities by one-way ANOVA and Tukey´s honestly significant difference (HSD) post hoc test. Correlation of reference gene Cq values was analyzed by Pearson's correlation coefficient.

## Results

### Identification and characterization of vHPOs

Nine different algae species were collected in the Baltic Sea between November 2020 and April 2021 (Table 1) and prepared for RNA sequencing. The obtained contigs were six-frame translated as a representation of the expressed proteome. As a basis for the identification of putative vHPOs by sequence homology, known vHPOs were extracted from the pdb database. Some of the entries correspond to highly related proteins, while sequence homology between others was extremely low. Therefore, vHPOs were subdivided into three groups (Fig. 1). Group 1 is constituted by vHPOs from the red alga *Corallina pilulifera*, the brown alga *Ascophyllum nodosum*, and the cyanobacterium *Acaryochloris marina*, group 2 from the bacterium *Zobellia galactanivorans*, and group 3 from the filamentous fungus *Curvularia inaequalis*. The catalytic center of these vHPOs is universal, supported by an excellent structural superposition and the presence of highly conserved residues for catalysis and vanadate-binding clustered in three regions referred to as motif 1 to 3 (Fig. 1). Due the overall low sequence homology between the groups, structural superposition was not reflected in the sequence-based alignments and thus the groups were kept separate for further analysis. The sequences of the known vHPOs were used in BLAST searches of the six-frame translated mRNA assemblies. Hits were classified as putative vHPOs, when the motifs were conserved. In total, approximately 70 putative vHPO sequences were obtained. All sequences have been deposited in NCBI GenBank database under the accession numbers from PQ699180 to PQ699250.

In candidate number 6 from *B. plumosa* (Bp_06), the histidine in motif 3 is replaced by asparagine. This is expected to render the protein catalytic inactive as this histidine residue forms a covalent bond to the vanadate. Thus, irrespective of the otherwise high sequence homology, this sequence is not expected to be a functional vHPO. Interestingly, in sequences originating from various algae assigned to group 2, the histidine in motif 2 is replaced by a serine or a threonine in 22 and 2 cases, respectively.

We used a pipeline of different bioinformatic tools to predict putative features of the enzymes encoded by the novel sequences (Supplementary Table S2). The molecular masses of complete enzymes range from 25.7 to 77.6 kDa and the isoelectric points from 4.3 to 9.8 (including potential signal peptides). Overall, a signal peptide was predicted in 50 instances, either by HECTAR (for brown algal sequences) or by SignalP, TargetP, and DeepLoc (for red and green algal sequences). Proteins with a predicted signal peptide are expected to enter the secretory pathway, with the majority predicted to be secreted into the extracellular space. Only in a few instances, the proteins may be located to the endoplasmic reticulum with moderate probability. Proteins lacking a signal peptide may remain in the cytoplasm or be located to the mitochondria or peroxisomes. The membrane probability is low for all proteins. A Kozak sequence (RxxATGG) was found in 20 instances. Peroxidase features were predicted for all submitted sequences by InterProScan.

**Fig. 2:**
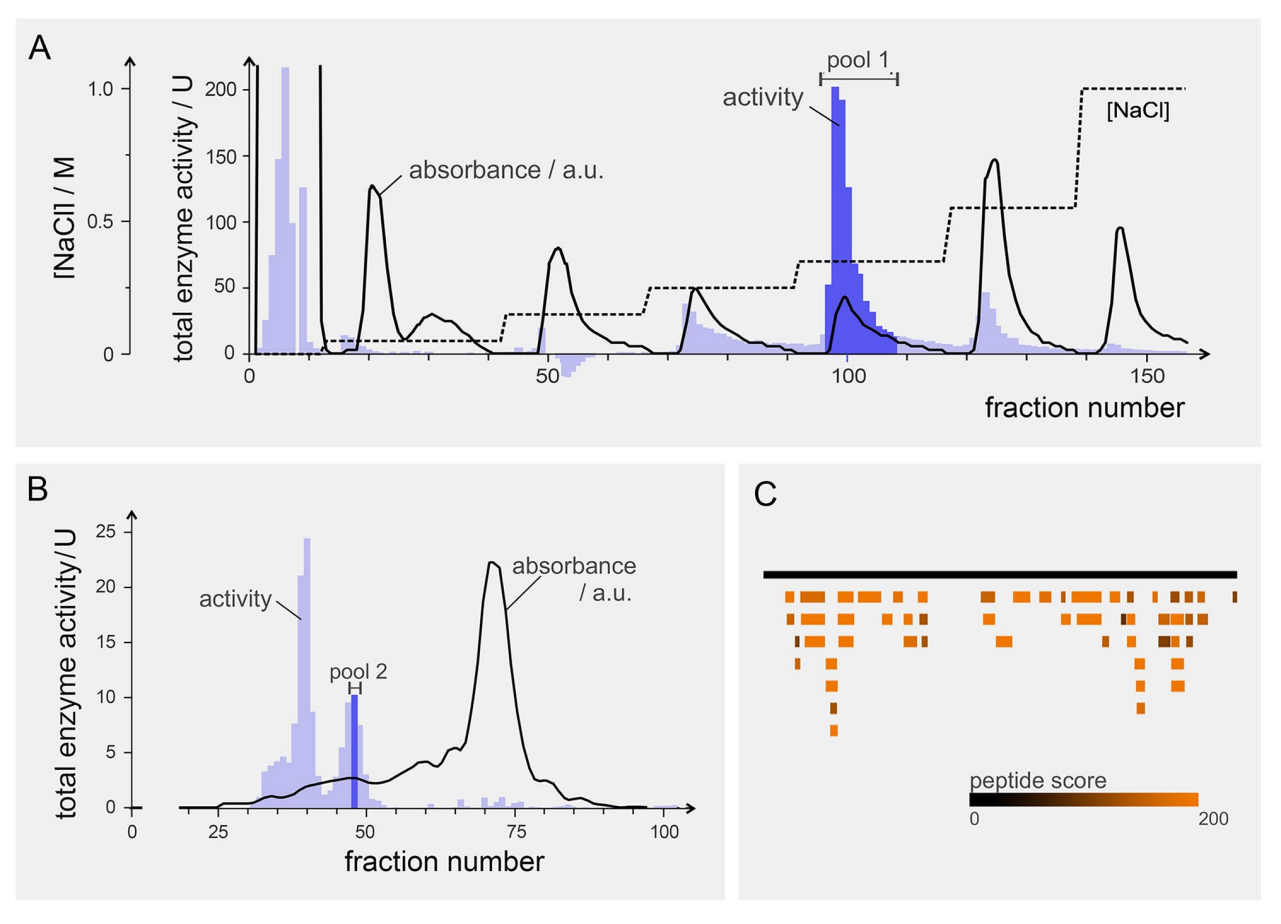
Purification of vHPO activity from *Saccharina latissima*. **A** Cell extract from *S. latissima* was separated by anion exchange chromatography with gradient elution as indicated. The fractions were assayed for peroxidase activity. The fractions with highest activity were pooled and concentrated (pool 1). **B** Pool 1 was further separated by size-exclusion chromatography. Fractions subjected to further analysis are labeled as pool 2 and correspond to an estimated molecular mass of approximately 150 kDa. The fractions were selected based on peroxidase activity, whereby fractions containing aggregated protein unable to enter native gels were excluded. **C** Peptide coverage of Sl1_29, a representative vHPO, that was identified by mass spectroscopy in a band visualized by in-gel assay for peroxidase activity from a 10% blue native polyacrylamide gel to which pool 2 was subjected. Identified peptides are colored by shades of red representing the peptide quality score as provided by the MaxQuant software package

For MS/MS analysis, a protein extract of *S. latissima* was fractionated by AEX and assayed for peroxidase activity (Fig. 2A). Fractions 96–108, which eluted with 350 mmol/L NaCl, exhibited the highest peroxidase activities and were thus pooled, concentrated, and subjected to further separation by SEC (Fig. 2B). Although fractions 39 and 40 demonstrated the greatest peroxidase activity, NP analysis indicated the formation of multiprotein complexes that were unable to migrate into the gel because of their size and shape (data not shown). Therefore, fractions 47–49 were subjected to NP of which the active band was cut and used for MS/MS analysis (Supplementary Fig. S1). Eight vHPOs, all clustered with the group 1 bromoperoxidase from *A. nodosum* (marked with asterisks in Fig. 1), were confidently identified in the active protein band of the *S. latissima* protein fractions. These hits exhibited excellent sequence coverage ranging from 60.4 to 80.5% and iBAQ scores between 1.5 × 10^6^ and 4.1 × 10^9^. From this group, we selected *Sl1_07*, *Sl1_29*, and *Sl1_31*—whose corresponding proteins were detected by MS—for stress experiments, along with *Sl1_41* from a separate cluster within the same vHPO group. While the cluster containing *Sl1_07*, *Sl1_29*, and *Sl1_31* was phylogenetically closely related to vanadium-dependent bromoperoxidases from *Saccharina japonica* and *Laminaria digitata*, the cluster including *Sl1_41* exhibited greater resemblance to their iodoperoxidase counterparts.

### Stress response

**Fig. 3:**
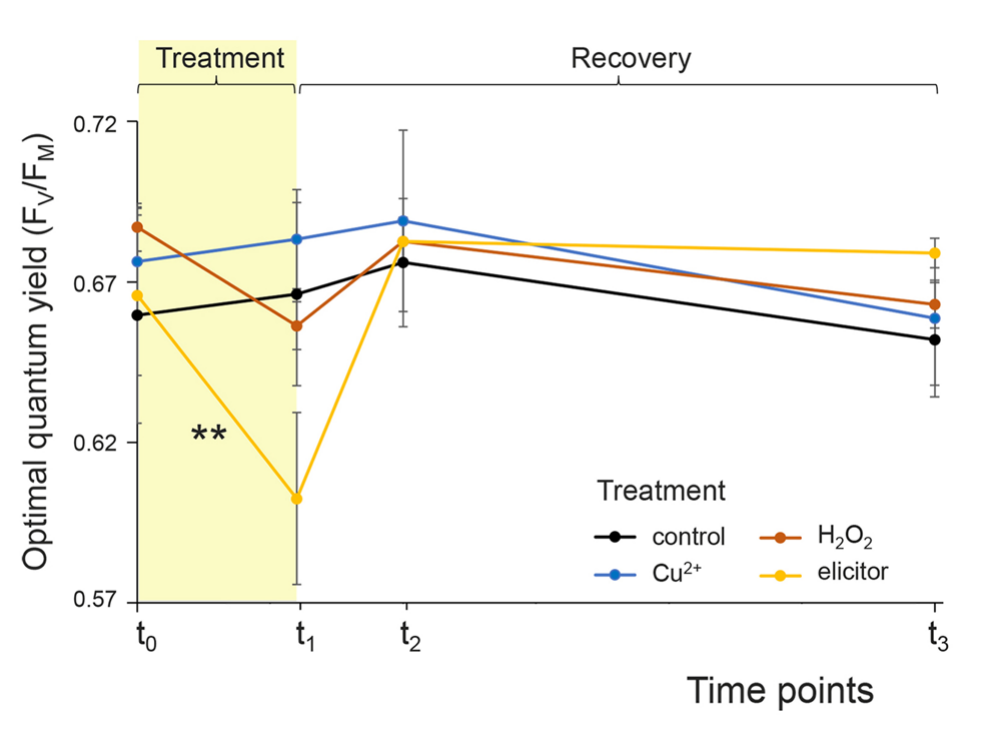
Optimal quantum yield (*F*_V_/*F*_M_) of *S. latissima* during the stress experiment. *t*_0_ = 6 h or 0.5 h before stress treatment, *t*_1_ = immediately after stress treatment, *t*_2_ and *t*_3_ = after a recovery time of 4 and 24 h, respectively. After each time point, half a thallus was removed per biological triplicate. *t*_0_: *n* = 6; *t*_1_: *n* = 6; *t*_2_: *n* = 3; *t*_3_: *n* = 3. Significant changes in *F*_V_/*F*_M_ between the time points were tested by one-way ANOVAs with subsequent Dunnett´s tests within the treatment groups, *P* < 0.01: **

By applying various stressors, the expression level of selected genes (*Sl1_07*, *Sl1_29*, *Sl1_31* and *Sl1_41*) in the algal stress response was assessed. To ensure that the alga is capable to cope with the applied stress, the concentrations of the respective stressors were determined in preliminary experiments using *F*_V_/*F*_M_ as an indicator of fitness (Supplementary Fig. S2). As higher concentrations of Cu^2+^ and H₂O₂ were found to cause irreversible damage to PS II, the stress intensities were carefully set below these levels to prevent permanent thallus damage. During the main stress induction experiment, the optimal quantum yield (*F*_V_/*F*_M_) of the thalli was determined prior to each treatment. Whereas the treatment with H_2_O_2_ and Cu^2+^ did not induce significant reductions of *F*_V_/*F*_M_ (Fig. 3), the elicitor treatment led to a mild but significant decline of *F*_V_/*F*_M_ of 10.6% ± 2.4% (one-sample *t*-tests, *µ* = 0 with a Bonferroni-corrected significance level of *α* = 0.0056 for nine conducted tests: *P* = 0.00019**) with a subsequent recovery to the initial level (Fig. 3). To confirm that this effect was not caused by any residual elicitor solution remaining on the thallus, test measurements were conducted on both the concentrated elicitor solution and a freshly immersed thallus (data not shown). While the algal tissue debris from the concentrated elicitor solution showed substantially reduced *F*_V_/*F*_M_, none of those particles remained on the thallus during measurement. Instead, normal *F*_V_/*F*_M_ data were recorded immediately after immersion, indicating that the measurement was not influenced by the elicitor solution in the seawater and that the stressed condition occurred during the incubation period. The control treatment group as well as the samples before each treatment (*t*_0_) revealed a mean *F*_V_/*F*_M_ of 0.67 ± 0.03 (mean ± sd), stating an unstressed condition for untreated thalli. Images from the Imaging-PAM fluorometer of all treated and untreated thalli of *S. latissima* are given in Supplementary Fig. S3.

We observed potential variations in the expression of the selected vHPO genes under stress conditions. Analysis of the fold change in gene expression was conducted in two different ways: (a) by normalizing each treated sample with the averaged Δ*C*q of the control group at the corresponding time point (fold change (to control)); (b) by normalizing each sample at *t*_1_ with the Δ*C*q of its paired sample *t*_0_ (fold change (to *t*_0_)).

**Fig. 4:**
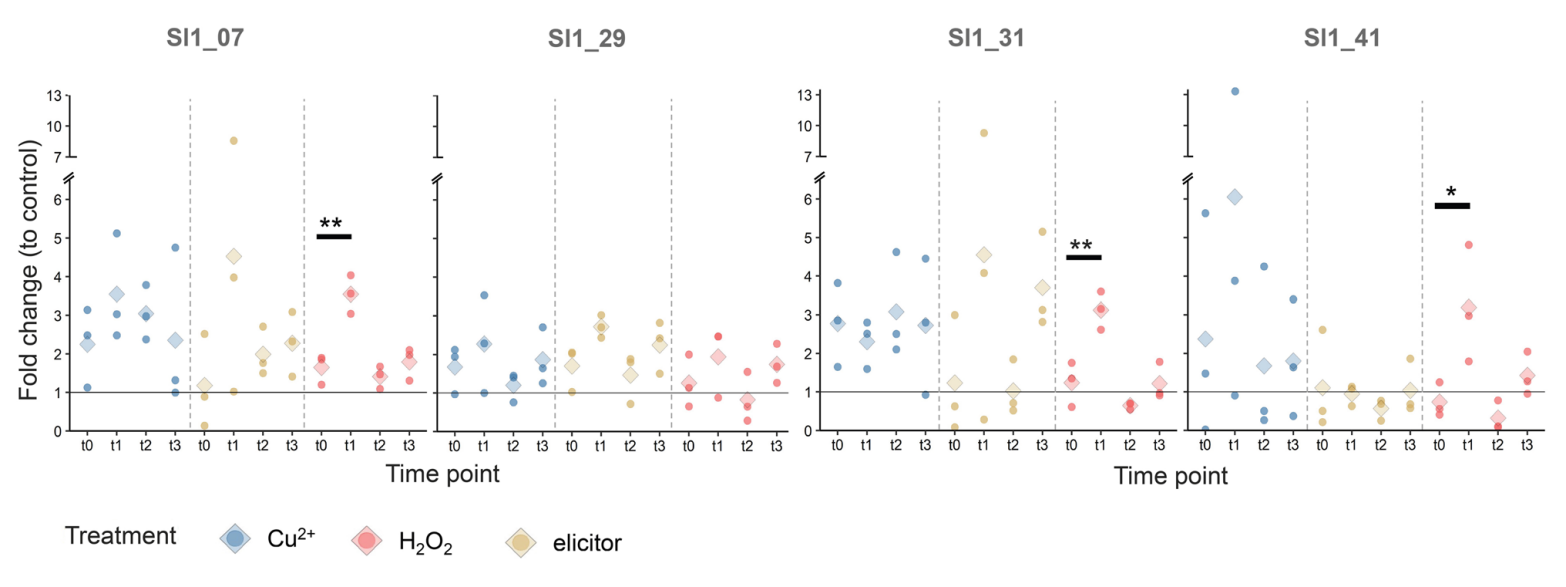
Fold changes (to control) of the genes of interest (*Sl1_29, Sl1_07, Sl1_31, Sl1_41*) in treated samples at time points *t*_0_, *t*_1_, *t*_2_ and *t*_3_ compared to the control group at the corresponding time point. A fold change of 1 indicates no change in gene expression compared to the control. To test for significantly altered gene expression, differences between the time points of each treatment group were tested by one-way ANOVA with subsequent Dunnett’s post hoc test using t_0_ as control. *P* < 0.05: *, *P* < 0.01: **. *n* = 3: biological triplicates (filled circles) with their average (diamond)

Fold change (to control) elucidated the evolution of gene expression over the course of the stress experiment (Fig. 4). Up-regulation was primarily observed directly after the treatments at time point *t*_1_, but less during the recovery phase at *t*_2_ and *t*_3_. Looking at the different treatments at *t*_1_, we found different responses to the different treatments: After H_2_O_2_ treatment, significantly increased transcript levels of *Sl1_07*, *Sl1_31*, and *Sl1_41* were observed, with mean fold changes ranging from 3.1 to 3.5 relative to the control. The elicitor treatment was associated with even higher fold changes of *Sl1_07* and *Sl1_31* at *t*_1_, reaching 4.5- and 4.6-fold, respectively, whereas expression of the putative vIPO gene *Sl1_41* remained unaffected. Similarly, Cu^2+^ treatment was associated with elevated gene expression, with the highest response observed for *Sl1_41* at *t*_1_, showing a 6.0-fold change relative to the control. However, none of the changes observed after the elicitor or Cu^2+^ treatment reached statistical significance due to low sample size and pronounced inter-individual variability.

**Fig. 5:**
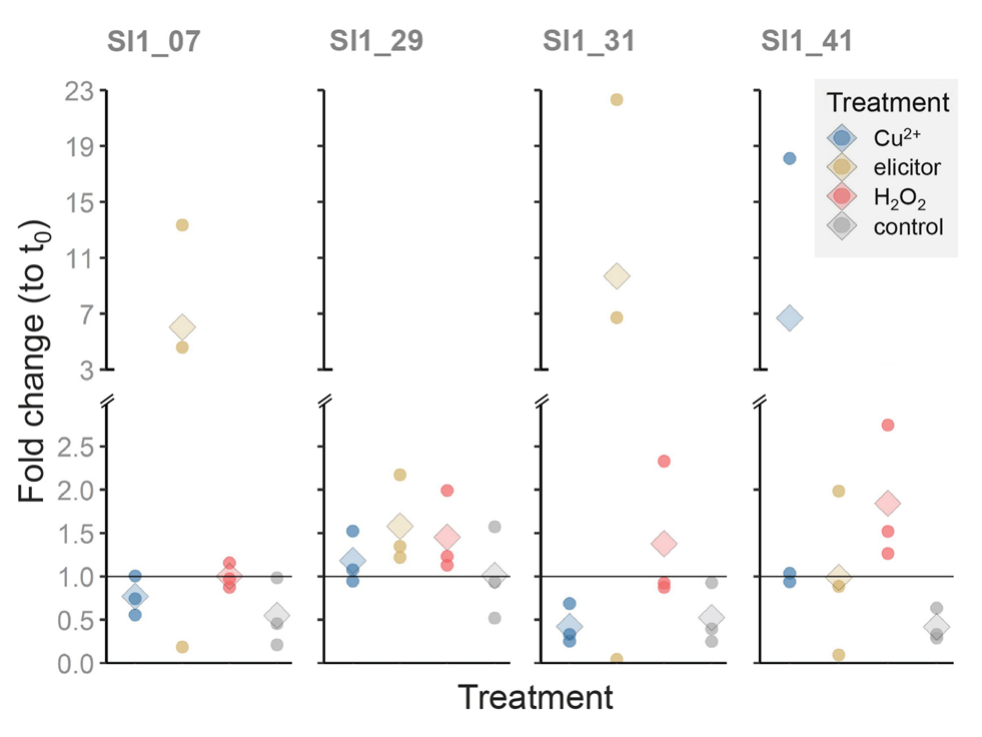
Fold changes (to *t*_0_) of the genes of interest (*Sl1_29, Sl1_07, Sl1_31, Sl1_41*) directly after stress application (time point *t*_1_) normalized by the corresponding sample at *t*_0_. Statistical differences between the treatment groups were tested by one-way ANOVA with subsequent Dunnett’s post hoc test comparing to the control group. Tests revealed no significant differences. *n* = 3: biological triplicates (filled circles) with their average (diamond)

To minimize the influence of this variability on treatment effect analysis, fold changes relative to *t*_0_ were calculated for *t*_1_ (Fig. 5). This analysis sets the paired thalli at *t*_0_ and *t*_1_ in relation and determines the change of gene expression prior and after stress on the same individual. Since the control treatment is not considered in this calculation, fold change (to *t*_0_) in the treatments was statistically compared to the control. The results showed similar tendencies to the results obtained with the fold change (to control) although no significant changes were observed. The pattern of the fold change (to *t*_0_) in elicitor-treated samples at *t*_1_ was the same as analyzed by fold change (to control), except that *Sl1_29*, was slightly less, and *Sl1_07* and *Sl1_31* were higher up-regulated with 1.6-, 6.0- and 9.7-fold change (to *t*_0_), respectively. The latter two were again characterized by high variability among triplicates. The H_2_O_2_-treated samples at *t*_1_ showed substantially lower fold changes (to *t*_0_) than fold changes (to control). Among these, *Sl1_41* showed the highest relative gene expression with a 1.9-fold change (to *t*_0_). After Cu^2+^ treatment at *t*_1_, the putative vBPOs showed nearly no up-regulation. Solely a single sample of the putative vIPO gene *Sl1_41* showed a 16-fold change (to *t*_0_), whereas the other two replicates showed no up-regulation.

## Discussion

### Novel vHPOs identified

We identified approximately 70 distinct novel vHPO sequences, which were shown to fall into three different vHPO groups. Structurally known enzymes of the first group from the red alga *Corallina pilulifera*, the cyanobacterium *Acaryochloris marina*, and the brown algae *Ascophyllum nodosum* represent bromoperoxidases, whereas representatives of the groups 2 and 3 from the marine bacterium *Zobellia galactanivorans* and the mold fungus *Curvularia inaequalis* represent iodo- and chloroperoxidases, respectively. Since the residues and motifs determining the most electronegative halide, which can be utilized by the enzyme are largely unknown (Fournier et al. 2014), we cannot definitively conclude whether the assignment to the corresponding groups provides information about their respective peroxidase type. In contrast, phylogenetic studies on vHPOs from marine algae have shown that vIPOs tend to cluster with vBPOs of related algae species, as they share a common ancestor (Colin et al. 2005). Therefore, we suspect that both bromo- and iodoperoxidases grouped with the bromoperoxidase of *Ascophyllum nodosum* and can be found in multiple clusters. Considering the high sequential similarity of vHPO sequences from *S. latissima* with bromo- or iodoperoxidases of other members of Laminariales (*Saccharina japonica*, *Laminaria digitata*), we conclude that the cluster from *Sl1_07* to *Sl1_38* encodes for vBPOs, whereas *Sl1_41* and *Sl1_46* are vIPO genes. Notably, multiple vHPOs from the vBPO cluster were identified through MS analysis in an active protein fraction of *S. latissima*. This finding provides evidence that these enzymes are indeed functional bromoperoxidases catalytically involved in bromination processes within the alga.

While the identified group 1 peroxidases exhibit higher similarity with bromo- and iodoperoxidases from other algae, sequences assigned to the groups 2 and 3 appear to be more distantly related to known vHPOs and may therefore be interesting targets for future studies. This is particularly the case for the group from *Ap1_10* to *Ap1_25*, whose corresponding protein sequences clustered exclusively with the vCPO of *Curvularia inaequalis*. However, as all algae samples were taken directly from the marine environment, a possible influence of epibionts cannot be excluded. To the best of our knowledge, vCPOs have not yet been described in algae.

Interestingly, in several novel sequences assigned to group 2, the histidine in motif 2—typically involved in vanadate coordination—is replaced by a serine or threonine (Fig. 1). This substitution has also been observed in certain vHPO enzymes from *Streptomyces* sp., such as NapH1 and Mcl24, which are both catalytically active and involved in the biosynthesis of halogenated natural products (Chen et al. 2022; McKinnie et al. 2018). The recurrence of such sequence variations may suggest that the spectrum of catalytic activities within the discovered sequences is broader than assumed. However, experimental validation is necessary to confirm this hypothesis.

### Stress response characteristics of vHPOs from *S. latissima*

The expression patterns of four identified vHPO genes in *S. latissima* were experimentally studied under various stress conditions to elucidate their roles in the macroalgae's stress response. Three of the four genes were up-regulated following the H_2_O_2_ treatment. Additionally, we found signs of up-regulation for two putative vBPOs after elicitor and one putative vIPO gene after Cu^2+^ treatment although strongly characterized by a high inter-replicate variability (Figs. 4, 5). The observed vHPO gene regulation supports the hypothesis that vHPOs are involved in managing environmental stress due to multiple mechanisms and functions, including the scavenging of reactive oxygen species (ROS) (Manley 2002; Ritter et al. 2010, 2014; Roeder et al. 2005), the facilitation of halogen uptake and accumulation (Küpper et al. 1998, 2008), the formation of halogenic compounds active against herbivores, pathogens and biofouling (Potin et al. 2002; Roeder et al. 2005; Wever et al. 2018), as well as the contribution to cell wall formation and strengthening (Roeder et al. 2005; Salgado et al. 2009). The subsequent sections will provide a detailed discussion of the individual regulation of gene expression of the discovered vHPO genes from *S. latissima* following the different stress treatments.

### Stress responses triggered by elicitor solution

Our data indicate that an elicitor treatment using a homogenized *S. latissima* tissue solution is capable of influencing gene expression in the alga: Enhanced gene expressions were detected for two of the three vBPOs directly after the elicitor treatment albeit strongly scattered und thus statistically not significant (Figs. 4, 5). However, a similar response to elicitors was observed in a transcriptome study on the brown alga *L. digitata* (Cosse et al. 2009). In addition to the potentially enhanced gene expression following the elicitor treatment, a physiological stress response was also observed. Here, a 10.6% reduction of *F*_V_/*F*_M_ was detected (Fig. 2). This finding is consistent with the well-documented ability of elicitors to cause rapid accumulation of H_2_O_2_ (oxidative burst; Küpper et al. 2001; Wojtaszek 1997), which in turn may diffuse into the chloroplasts and create damage on the photosynthetic machinery (Ashraf and Harris 2013). Induction with elicitors triggered oxidative burst in the brown alga *L. digitata* has been obtained after exposure to enzymatically or chemically prepared oligosaccharides derived from alginate (Cosse et al. 2009; Küpper et al. 2001). In this context, Küpper et al. (2001) suggested a strong dependency of the molecular structure of oligosaccharides on its elicitation effect. Although *S. latissima* contains 20–30% alginate in its dry weight (Schiener et al. 2015), it is questionable whether the mechanical homogenization of the thalli used as the elicitor solution in this experiment produced such molecular fragments. Nevertheless, the results of this study suggest that the applied elicitor solution was potent for the induction of a stress response. The algae fully recovered within four hours after stress-based reduction of *F*_V_/*F*_M_ (Fig. 3), indicating that the alga is capable to cope with the stress induced by this treatment. This phenomenon may be attributed to a rapid onset of the oxidative burst, followed by efficient scavenging of reactive oxygen species (ROS) by antioxidants. Similarly, recovery of *F*_V_/*F*_M_ was observed after H_2_O_2_ stress in *Ectocarpus siliculosus* (Dittami et al. 2009).

### Minor stress responses due to H_2_O_2_ treatment

Since it is well-established that the application of elicitors leads to the accumulation of H_2_O_2_ in plant tissues (Küpper et al. 2001; Wojtaszek 1997), it was assumed that the effect of elicitors might be similar to the effects of direct H_2_O_2_ treatment. However, in contrast to the elicitor treatment, the application of 3 mmol/L H_2_O_2_ did not substantially affect *F*_V_/*F*_M_ in *S. latissima* (Fig. 3; Supplementary Fig. S2). This also stands in contrast to the observation made in *E. siliculosus*, where even 1 mmol/L H_2_O_2_ treatment induced a strong reduction in *F*_V_/*F*_M_ (Dittami et al. 2009). As the anatomy of an organism has an impact on stress resistance (Koyro and Huchzermeyer 2018), one could suspect that *E. siliculosus* is more susceptible to a surrounding stressor due to its filamentous morphology increasing the relative surface in comparison to the compact parenchymatous thallus of *S. latissima*. In a comparable study on parenchymatous Phaeophyceae, 1 mmol/L H_2_O_2_ had no effect on *F*_V_/*F*_M_, but 5 mmol/L led to a clear reduction (Dummermuth et al. 2003). Possibly, the applied 3 mmol/L H_2_O_2_ set a stress intensity too low to affect *F*_V_/*F*_M_ in *S. latissima*. Due to its high oxidizing potential (Petrov and van Breusegem 2012), the amount of H_2_O_2_ added to the seawater may have rapidly declined before reaching the chloroplasts, and the plant's own antioxidant system may have been involved in H_2_O_2_ scavenging before PS II damage occurred. In addition, damaged PS II may have been repaired by an active repair cycle of PS II (Takahashi and Murata 2008).

Even though the applied H_2_O_2_ did not affect the function of PS II, the results of this study indicate that the applied concentration was yet effective to influence gene expression. Enhanced gene expression was detected for two of the three vBPOs directly after the treatment at *t*_1_ although this enhancement was more pronounced when analyzed in comparison to the control sample rather than to *t*_0_ (Figs. 4, 5). The observed elevation in gene expression is consistent with the study on the brown alga *E. siliculosus* that showed a 1.6-fold enhancement of its vBPO upon 1 mmol/L H_2_O_2_ treatment (Dittami et al. 2009). To the best knowledge of the authors, gene expression of vHPOs in response to H_2_O_2_ treatment has not been studied in sporophytes of Laminariaceae. However, oxidative stress induced by isolation of protoplasts enhanced gene expression of various vBPOs in *L. digitata* (Roeder et al. 2005). Notably, these vBPOs were verified to be also present in sporophytes. However, while neither of the two aforementioned studies found a stress-induced up-regulation of vIPO, in this present study the putative vIPO was found to be 1.9- or 3.1-fold elevated in response to H_2_O_2_.

### Cu^2+^ treatment potentially affects vIPO stress responses

In contrast to elicitor and H_2_O_2_ treatment, neither of the assessed parameters *F*_V_/*F*_M_ nor gene expression were found to be clearly affected by the six-hour treatment with 300 µg/L Cu^2+^. This observation was made despite literature suggesting severe damage upon Cu^2+^ treatment as indicated for instance by lipid peroxidation in *L. digitata* after 24 h (140 µg/L Cu^2+^, Ritter et al. 2008) or bleaching at the meristem of *S. japonica* after three days (200 µg/L Cu^2+^, Zhang et al. 2019). In the latter study, *F*_V_/*F*_M_ was also not found to be reduced. However, a distinct reduction of *F*_V_/*F*_M_ was observed in *E. siliculosus* upon six hours of 500 µg/L CuCl_2_ corresponding to 238 µg/L Cu^2+^ (Ritter et al. 2014). Again, the morphological differences of the algae may be responsible for a higher susceptibility of *E. siliculosus* to this stressor. On the level of gene expression, Ritter et al. (2014) found an up-regulation of the vBPO of *E. siliculosus* as a response to this Cu^2+^ treatment. In *S. latissima*, copper stress has not been studied. In the morphologically similar species *S. japonica*, four- and six-fold upregulations of vBPOs upon treatment with 100 and 200 µg/L Cu^2+^ were observed, respectively, when applied for 3 days (Zhang et al. 2019). Therefore, applying 300 µg/L Cu^2+^ for 6 h was putatively either too low or too short to influence *F*_V_/*F*_M_ or the transcription level of the tested putative vBPOs in *S. latissima*. Nonetheless, gene expression of the putative vIPO was up-regulated six-fold although this resulted from exceptionally high gene expression in one of the three replicates (Figs. 3, 4). Hence, the biological variability was too high to claim this result with statistical certainty considering the limited sample size. Given that the regulatory expression of vIPO genes in response to Cu^2+^ treatment has not been explored previously, further examination of this result is of considerable interest.

### Methodological considerations

Overall, the gene expression data were dominated by a large heterogeneity of *S. latissima* individuals (Supplementary Fig. S4). The individuals were collected in the field. Although grown in the same location, they could have emerged from very different genetic origins (Schiel and Foster 2006) and been exposed to different environmental constraints. Hence, laboratory-cultured sporophytes could be used for further investigation, which have a more uniform genotype and were grown under controlled conditions. The ΔΔ*C*q method relies on stability of untreated calibrator samples, with which the treated samples are normalized (Livak and Schmittgen 2001). The experimental design of this study contained two types of untreated calibrator samples that can be used for this purpose: the control group, which was run in parallel to the treatments, and the t_0_ samples that were sampled in each treatment group prior to the treatment.

Our data suggest an impact of the calibrator sample selection on the outcome. As the variation in the qPCR data was largely influenced by biological variation, the use of the paired samples as calibrators was considered to be more accurate for determining a stress response in the treated individuals. The paired samples for pre–post-analysis were generated with a longitudinal cut through the algal thallus, producing two bilaterally identical halves with a meristem rich in regulatory gene expression (Zhang et al. 2019). However, this method limits paired analysis to two time points. Furthermore, the longitudinal sectioning of the thalli may have impacted the stress tolerance. Even though no significant changes in optimal quantum yield were observed for the cut control thalli, which states unstressed conditions, stress can manifest at another site of the plant that does not influence photosynthesis and thus cannot be measured as a change in *F*_V_/*F*_M_ (Murchie and Lawson 2013). Due to the high biological variation, a larger sample size is recommended in future experiments to enhance the statistical power.

## Conclusion

In conclusion, this study contributes to the understanding of vHPOs from macroalgae and provides novel vHPO sequence data from nine different algal species. The workflow used in this study can serve as a guideline for the biodiscovery of enzymes, especially peroxidases, from algae. Our results provide insights into potential vHPO-related stress response mechanisms of *S. latissima* under different stress conditions. The results suggest that these enzymes may play a role in mitigating oxidative stress, as evidenced by their up-regulation upon H_2_O_2_ treatment. However, the data of the response to elicitor and Cu^2+^ stress were less conclusive as the overall data were characterized by a large inter-replicate variation, indicating the need for further investigation.

## Supplementary Information

Below is the link to the electronic supplementary material.

Supplementary file1 (PDF 1072 KB)

Supplementary file2 (XLSX 89 KB)

## Data Availability

Data supporting the findings of this study are available from the corresponding author upon reasonable request.
