# Supplementary material for: Novel vanadium-dependent haloperoxidases from macroalgae and their expression in response to biotic and abiotic stressors in Saccharina latissima
Source: Mar Life Sci Technol. 2026 Mar 30;8(2):404–18. doi: 10.1007/s42995-026-00367-4 (PMC13198595; doi:10.1007/s42995-026-00367-4)
Supplement: Supplementary file 1 — Supplementary file1 (PDF 1072 KB) [file 42995_2026_367_MOESM1_ESM.pdf]

Supplementary

Table S1 Specific primer used for reverse transcriptase quantitative polymerase chain reaction (RT-qPCR) analysis of gene expression

Gene	Primer sequence (5′–3′)	Product length (bp)
Sl1_07	Fwd: TACCCCAACCCCGTAGTCTC<br>Rev: GGTAAGGCCGGTGGTCTTTT	120
Sl1_29	Fwd: ACTTCTGTGACGAGGTGCAG<br>Rev: AGTGTGGAGCGAGAACTTGG	83
Sl1_31	Fwd: GTGCCGACAAAGCACGAAAT<br>Rev: GATGTGCGACCTTCCCAAGA	126
Sl1_41	Fwd: TTCAACCTGTTCAGTGGCGA<br>Rev: TGGCCCCTTCAAACAACACT	97
EIF5B	Fwd: GAGGTGACGAGCGATTGA<br>Rev: TCTTTCGGCGACGGGTT	107
NDH	Fwd: GGAGGAGATGGAGGAGCGTGTG<br>Rev: GGCGTCCGTGCGGTAGAAGT	166

Table S2: available in supplementary file

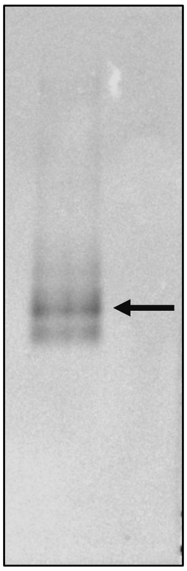

Fig. S1 In-gel peroxidase activity of *S. latissima* protein fractions from chromatographic purification (pool 2 from SEC). Fractions with in-solution peroxidase activity were separated by 8% native PAGE. In-gel peroxidase activity was visualized using thymol blue staining in the presence of H_2O_2 and KBr. The arrow indicates the excised active band subjected to mass spectrometric analysis.

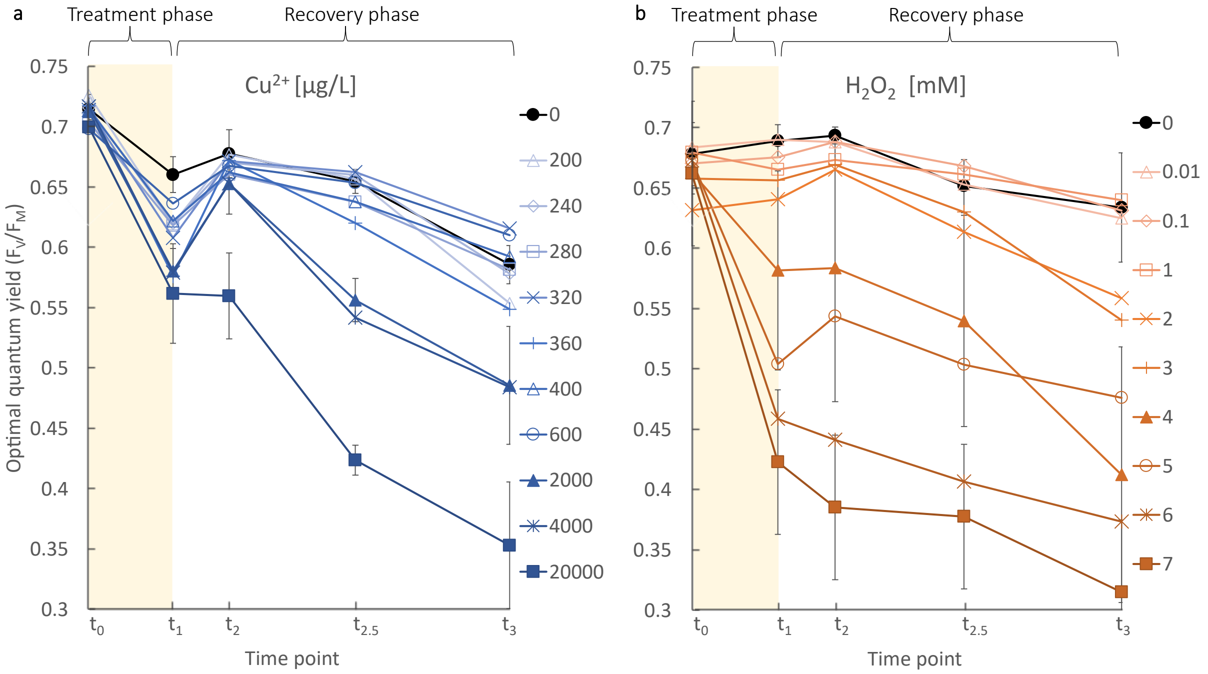

Fig. S2 Effect of different concentrations of Cu^{2+} in µg/L (**a**) and H_2O_2 in mmol/L (**b**) on the optimal quantum yield (F_V/F_M) in 2 cm x 2 cm pieces of *S. latissima*. Sample size per treatment group: n = 5. For reasons of clarity, error bars are given exemplarily for three samples (filled symbols) only, representing approximately the size of the standard deviations of the other treated samples. F_V/F_M of the control groups revealed a general decrease with time. t_0: 6 h or 0.5 h before Cu2+-treatment or H_2O_2-treatment, respectively; t_1: immediately after stress treatment; t_2, $t_{2.5}$ and t_3: after a recovery time of 4, 13 and 24 hours, respectively

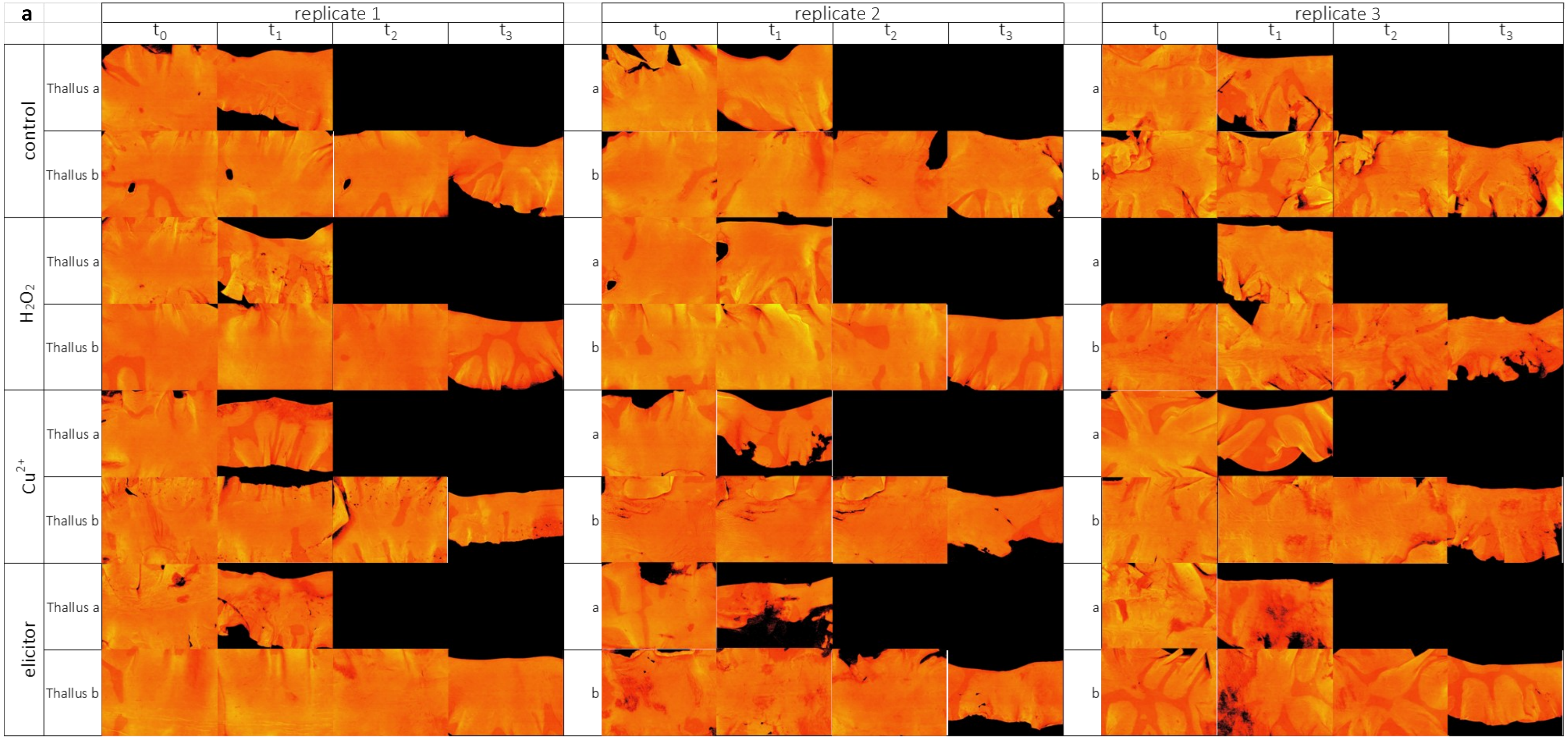

a
replicate 1
replicate 2
replicate 3
t0
t1
t2
t3
control
H2O2
Cu2+
elicitor
Thallus a
Thallus b
a
b

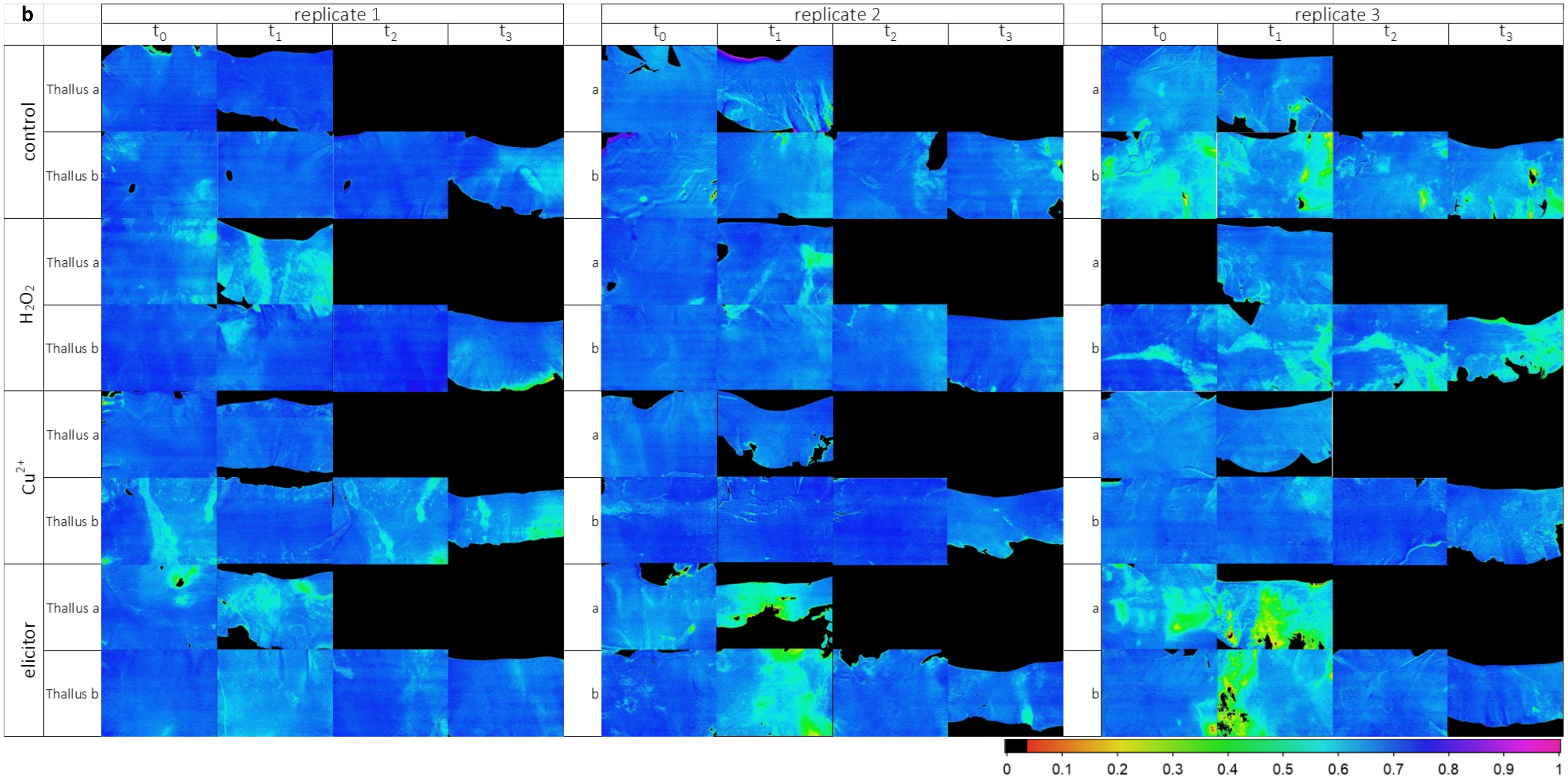

Fig. S3 Fluorescence measurements assessed as F_0 (**a**) and F_V/F_M (**b**) using an Imaging-PAM fluorometer of thalli of *S. latissima* subjected to different treatments. Each treatment group consisted of two thalli (thallus a and b). After each time point one half of a thallus (first from thallus a, then from thallus b) was cut longitudinally and removed from the experiment. Thus, thalli at time point t_1 and thallus b at time point t_3 is halved. Fluorescence values are visualized in color as indicated by the scale below the panels. The thalli were placed in the direction of growth from left to right and randomly faced upwards. All measurements were taken for three biological replicates. Replicate 3 of the H_2O_2 treatment at t_0 is missing. Size of each panel: 15 cm x 13 cm

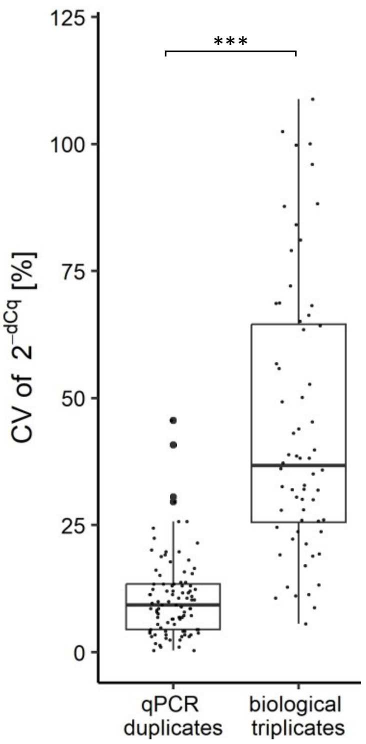

Fig. S4 Sources of variability within ΔCq values (computed as linearized $2^{-\Delta Cq}$) of the genes of interest, presented as their coefficient of variation (CV, in %). Shown are the CV of all qPCR duplicates (n = 96) and of all biological triplicates (n = 64, averaged over the qPCR duplicates). Groups were statistically tested by Mann-Whitney-U test, P <0.001: ***
